# Molecular monitoring of *Plasmodium falciparum* super-resistance to sulfadoxine–pyrimethamine in Tanzania

**DOI:** 10.1186/s12936-016-1387-2

**Published:** 2016-06-23

**Authors:** Reginald A. Kavishe, Robert D. Kaaya, Sidsel Nag, Camilla Krogsgaard, Jakob Ginsbak Notland, Adellaida A. Kavishe, Deus Ishengoma, Cally Roper, Michael Alifrangis

**Affiliations:** Kilimanjaro Christian Medical University College, Moshi, Tanzania; National Institute for Medical Research, Tanga Centre, Tanzania; Centre for Medical Parasitology, Department of International Health, Immunology & Microbiology, Faculty of Health and Medical Sciences, University of Copenhagen, Copenhagen, Denmark; London School of Hygiene and Tropical Medicine, London, UK

**Keywords:** *Plasmodium falciparum*, Sulfadoxine-pyrimethamine, SP-resistance, SP-super resistance, Anti-malarial drugs, Tanzania, Drug resistance, Malaria, Mutations, Parasites, Polymorphisms

## Abstract

**Background:**

Sulfadoxine–pyrimethamine (SP) is recommended for prophylactic treatment of malaria in pregnancy while artemisinin combination therapy is the recommended first-line anti-malarial treatment. Selection of SP resistance is ongoing since SP is readily available in health facilities and in private drug shops in sub-Saharan Africa. This study reports on the prevalence and distribution of *Pfdhps* mutations A540E and A581G in Tanzania. When found together, these mutations confer high-level SP resistance (sometimes referred to as ‘super-resistance’), which is associated with loss in protective efficacy of SP-IPTp.

**Methods:**

DNA samples were extracted from malaria-positive blood samples on filter paper, used malaria rapid diagnostic test strips and whole blood collected from eight sites in seven administrative regions of Tanzania. PCR–RFLP and SSOP-ELISA techniques were used to genotype the A540E and A581G *Pfdhps*. Data were analysed using SPSS version 18 while Chi square and/or Fischer Exact tests were used to compare prevalence between regions.

**Results:**

A high inter-regional variation of *Pfdhps*-540E was observed (χ^2^ = 76.8, p < 0.001). High inter-regional variation of 581G was observed (FE = 85.3, p < 0.001). Both Tanga and Kagera were found to have the highest levels of SP resistance. A high prevalence of *Pfdhps*-581G was observed in Tanga (56.6 %) in northeastern Tanzania and in Kagera (20.4 %) in northwestern Tanzania and the 540–581 EG haplotype was found at 54.5 and 19.4 %, respectively. *Pfdhps*-581G was not detected in Pwani and Lindi regions located south of Tanga region.

**Conclusions:**

Selection of SP super-resistant *Pfdhps* A581G is highest in northern Tanzania. Variation in distribution of SP resistance is observed across the country: northeastern Tanga region and northwestern Kagera region have highest prevalence of SP super-resistance markers, while in Pwani and Lindi in the southeast the prevalence of super-resistance was zero. More studies should be conducted to understand the factors underlying the remarkable heterogeneity in SP resistance in the country.

## Background

Emergence and spread of *Plasmodium falciparum* resistance to chloroquine (CQ) and sulfadoxine-pyrimethamine (SP) forced the adoption of artemisinin-based combination therapy (ACT) as first-line anti-malarial drugs in most sub-Saharan African countries (SSA) by 2007 [1]. Since the introduction of ACT, SP has remained the combinational drug for ACT (SP-artesunate) used in a few SSA countries, or as a prophylactic drug in intermittent preventive treatment of malaria during pregnancy (IPTp) where three or more treatment doses of SP are administered on a monthly basis after the first trimester. SP as a prophylaxis is also used in infancy (IPTi) and as a seasonal malaria chemoprevention (SMC) in children [2–4]. SMC in particular, where SP is combined with amodiaquine, is currently being implemented in West African countries (Chad, The Gambia, Niger, Mali, Senegal, Guinea) [5], and it has been shown to be cost effective in reducing childhood morbidity and mortality due to malaria [6].

SP acts by inhibiting the folic acid synthesis in the malaria parasite; sulfadoxine and pyrimethamine inhibit the *Plasmodium falciparum* enzymes dihydropteroate synthetase (DHPS) and dihydrofolate reductase (DHFR), respectively [7, 8]. Resistance to sulfadoxine and pyrimethamine is caused by mutations in the *P. falciparumdhps* and *dhfr* genes, respectively. *Pfdhfr* single-point mutations causing amino acid changes in N51I, C59R and S108 N are the most common mutations associated with pyrimethamine resistance in SSA [9, 10] and the combination of these forming the triple *Pfdhfr* IRN mutation is highly prevalent in SSA [11, 12]. Regarding sulfadoxine resistance, *Pfdhps* mutations S436A/C/F, A437G, K540E, A581G, and A613S/T have been observed globally [13, 14] and S436A/C/F, A437G and G540E are commonly observed in SSA [11]. The combination of the *Pfdhfr* triple and the *Pfdhps* double (A437G, K540E) mutations collectively form the quintuple mutations [7, 15] which confers high-level SP resistance and is a significant predictor of SP *P. falciparum* treatment failure [16–18]. The evolution of the *Pfdhps* mutations normally occurs after the *Pfdhfr* triple mutations and thus the presence of *Pfdhps* double mutations indicates presence of quintuple mutations.

In SSA, the highest prevalence of SP resistance markers has been documented in East Africa where the quintuple mutation has been shown to approach fixation [12]. Evidence shows that the quintuple mutation emerged in the 1990s and in Tanzania as high as over 60 % was detected in 1998 [19], while a recent survey documented more than 95 % quintuple mutation, in 2011 [20]. In 2010, the World Health Organization (WHO), through its malaria advisory committee made a recommendation that SP-IPTi should be implemented only in areas where prevalence of the quintuple mutation, as represented by the *Pfdhps* 540E, is less than 50 % [21]. Given the high prevalence of the 540E in East Africa, this policy has led to the restricted recommendation of SP-IPTi to West Africa where the 540E mutation (and hence the quintuple mutation) is low or absent [11]; currently, SP-IPTi is only implemented in Chad [5].

In areas where the quintuple mutation is high, IPTp with SP does not prevent placental malaria but may continue to protect against severe pregnancy outcomes [22–25]. WHO has continued to recommend use of at least three SP-IPTp doses even in such areas where the quintuple mutations are high [26].

The emergence of an additional *Pfdhps* mutation that is 581G in areas where the quintuple mutant is well established has been documented in two major foci in East Africa [12]. Furthermore, in Tanzania in particular, the prevalence of the 581G has reached 55 % in the northeast [27]. Growing evidence from studies performed mainly in Tanzania indicates that presence of the *Pfdhps* 581G (sextuple mutant parasites), is associated with reduced SP-IPTp efficacy by: (1) a reduction in the protection period of SP-IPTp from 4 to 2 weeks [23]; (2) increased parasitaemia [23, 28] and recently, from a study in Malawi [29], also increased placental parasitaemia [28]; (3) increased risk of severe malaria in offspring [30]; and, (4) low birth weight in new-borns from mothers undergoing SP-IPTp in Tanzania [31]. Thus, the emergence of sextuple mutants seems to have a direct impact on the efficacy of the IPTp using SP. The existing information on the 581G distribution in Tanzania is scarce, mainly confined to the region of Tanga, and may not be evenly distributed across the country. A recent systematic review and meta-analysis of the influence of the *Pfdhps* 581G mutation on SP-IPTp has shown that when the prevalence of 581G is >10 %, IPTp with SP does not protect against low birth weight [32]. For a proper, evidence-based implementation of IPTp programme and an understanding of regions where the strategy may be compromised, a countrywide monitoring of the mutations is important. This study reports on the status of *Pfdhps* K540E and A581G in Tanzania.

## Methods

Filter paper blood spot, whole blood and used malaria rapid diagnostic test strips collected in previous studies between June 2010 and August 2011 in seven regions of mainland Tanzania were used for this study, as previously described [33]. The study sites included Mwanza (Misungwi district) and Kagera (Muleba district) around Lake Victoria in the northwestern zone, Tanga (Muheza and Bondo) in the northeastern zone, Mtwara (Tandahimba and Mtwara-Urban), Coastal Region (Kibiti-Rufiji) and Lindi Region (Nachingwea) in the southeastern zone and Mbeya (Kyela and Rungwe districts) in the southwestern zone. The DNA samples were extracted using Chelex-100 method [34]. Genotyping for *Pfdhps* K540E and A581G was performed using polymerase chain reaction-restriction fragment length polymorphisms (PCR-RFLP) and polymerase chain reaction-single strand oligonucleotide probes-enzyme linked immunosorbent assay (PCR-SSOP-ELISA) methods described by others [35–37]. All PCR reagents and restriction endonucleases were purchased from New England Biolabs (Ipswich, MA, USA). Primers were purchased from Biolegio (Nijmegen, The Netherlands). Prevalence was calculated for each genotype, excluding mixed infections in both individual genotype prevalence and in 540-581 haplotype analysis. Only a few mixed infections were observed in A581G (Table 1). Maps were constructed using ArcGIS version 10.2. The study received ethical approval from the Kilimanjaro Christian Medical University College Research Ethics Review Committee.

**Table 1:** Distribution of *Pfdhps* K540E and A581G polymorphisms among regions in Tanzania

		540				581				Haplotypes				
Region	Site	K	E	K/E	Total (n)	A	G	A/G	Total (n)	KA	KG	EA	EG	Total (n)
Mwanza	Misungwi	4 (3.0)	129 (97.0)	0	133	118 (95.2)	6 (4.8)	0	124	4 (3.2)	0 (0.0)	114 (91.9)	6 (4.8)	124
Mbeya	Multiple sites	3 (2.0)	150 (98.0)	0	153	143 (93.5)	9 (5.9)	1 (0.7)	153	3 (2.0)	0 (0.0)	140 (92.1)	9 (5.9)	152
Mtwara	Tandahimba and Mtwara urban	30 (36.1)	53 (63.9)	0	83	76 (95.2)	4 (4.8)	0	80	29 (34.9)	1 (1.2)	50 (60.2)	3 (3.6)	83
Lindi	Nachingwea	24 (27.3)	64 (72.7)	0	88	88 (100)	0	0	88	24 (27.3)	0 (0.0)	64 (72.7)	0 (0.0)	88
Tanga	Bondo	10 (8.6)	106 (91.4)	0	116	46 (40.6)	64 (56.6)	3 (2.7)	113	7 (6.4)	4 (3.6)	39 (35.5)	60 (54.5)	110
	Muheza	5 (5.7)	83 (94.3)	0	88	43 (48.9)	45 (51.1)	0	88	5 (5.7)	0 (0.0)	38 (43.2)	45 (51.1)	88
Pwani	Rufiji	23 (23.7)	74 (76.3)	0	97	100 (100)	0	0	100	23 (23.7)	0 (0.0)	74 (76.3)	0 (0.0)	97
Kagera	Muleba	8 (4.7)	162 (95.3)	0	170	86 (79.6)	22 (20.4)	0	108	4 (3.7)	1 (0.9)	82 (75.9)	21 (19.4)	108
Total		107	821	0 (0)	928	700	150	4	854	99 (13.2)	6 (0.6)	601 (70.1)	144 (16.0)	850

Data are given as sample size and prevalence (%) in brackets

## Results

### *Pfdhps* K540E and A581G distribution

**Fig. 1:**
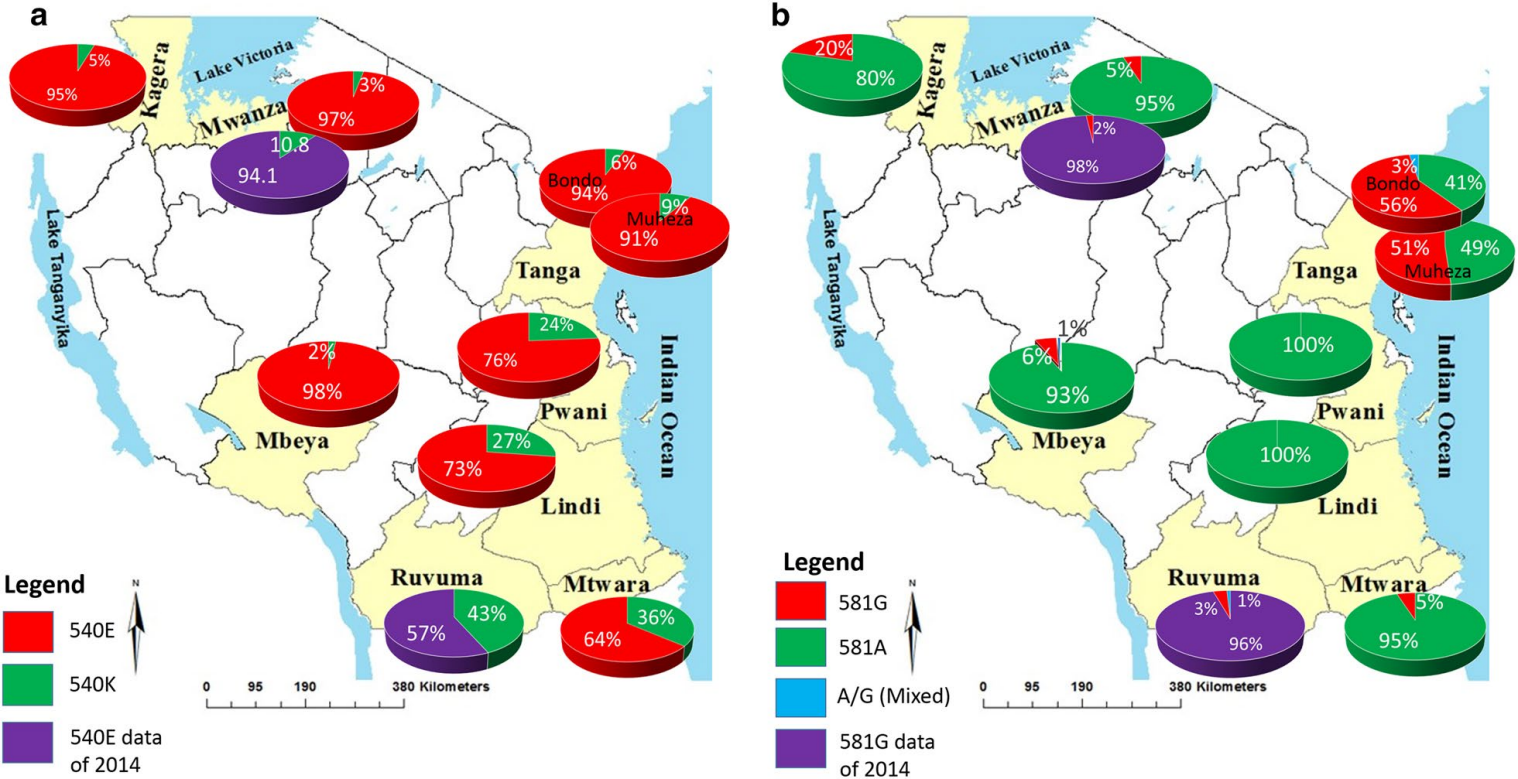
Regional sites and distribution of *Pfdhps* polymorphisms in Tanzania. **a** *Pfdhps* K540E and **b** *Pfdhps* A581G. Mutants are shown in *red* and wild types in *green*. Mixed genotypes are shown in *light blue*. All samples were collected in 2010/2011 except data shown in *purple*. Shown in* purple* is preliminary data for samples collected in 2014 for Mwanza and Ruvuma regions where similarity in *Pfdhps* 540E and 581G prevalence between Ruvuma and neighbouring Mtwara region is observed

A total of 1024 samples were available for the study from eight sites in seven regions; 928 and 854 were successfully genotyped for *Pfdhps* K540E and A581G, respectively. Genotypes are summarized in Table 1. The prevalence of the 540E mutation was generally high in all regions with the highest observed in Mbeya (98.0 %) and the lowest in Mtwara (63.9 %). Mtwara, Lindi and Pwani regions had significantly lower prevalence of 540E (average 71.3 %) compared to the rest of the country (average 92.4 %) (χ^2^ = 76.8, p < 0.001). For the 581G mutation, Muheza and Bondo sites in Tanga region had the highest prevalence at 51.1 and 56.6 %, respectively, followed by Kagera region (Muleba) where 20.4 % was recorded. Contrarily, the 581G mutation was absent in Pwani and Lindi regions and, again by comparing the southeast Mtwara, Lindi and Pwani regions against the rest of the country, only 1.5 % of the 581G was recorded in these three regions compared to 22.6 % in the rest (FE = 85.3, p < 0.001) (Table 1; Fig. 1).

### *Pfdhps* haplotypes

Construction of *Pfdhps K*540E-A581G haplotypes was performed and is shown in Table 1. The most prevalent haplotype was the EA ranging from 35.5 to 92.1 %. This is due to the high prevalence of the 540E as opposed to the 581G. The double mutant EG haplotype was most prevalent in Tanga, both in Muheza (51.1 %) and Bondo (54.5 %); it was observed at 3.6 % in Mtwara and 19.4 % in Kagera, while being absent in Pwani and Lindi.

## Discussion

Historically, pyrimethamine has been in use in Tanga region since the 1950s [38]. In 1954 resistance to a weekly prophylactic dose of pyrimethamine was reported in Tanga and this was the first resistance report in Tanzania and in Africa [39]. SP continued both as the second-line treatment drug [chloroquine (CQ) was the first-line drug] and for malaria prophylaxis until 2001, when it was declared the first-line drug due to high levels of CQ resistance [40]. However, since resistance to SP had already emerged, at least in some parts of the country, SP policy in 2001 was considered a temporary solution [41] and was replaced by ACT by 2006 [42].

In the East African region, the prevalence of molecular markers of SP resistance has been increasing since the emergence of the first resistance-conferring mutations in the 1950s. During the past decade, many of these mutations in *Pfdhfr* and *Pfdhps* have approached fixation levels, while newer mutations are still increasing [20, 27, 43, 44]. Given the importance of preventing malaria in pregnancy, the growing evidence of declining effectiveness of the IPTp strategy in areas of high prevalence of quintuple and sextuple *Pfdhfr/Pfdhps* haplotypes and the lack of an alternative to SP for use in IPTp, then the monitoring of SP resistance is crucial [45]. This is the first multi-site study in Tanzania to report on countrywide prevalence of these markers of SP resistance of immediate importance. The *Pfdhps* A581G mutation was first reported at low prevalence of 1.6 and 1.2 % in Mlimba (Morogoro) and Matema (Mbeya), respectively, in 2005 [46]. Surveys done in Tanga (Hale) in 2006 and 2007 found the mutation at prevalence of up to 54 % [27, 47]. In this study, Tanga region represents the highest prevalence of *Pfdhps* 581G followed by Kagera region. Highest levels of molecular markers of resistance (or tolerance) to other anti-malarial drugs, such as CQ, have also been recorded in Tanga when compared to other regions in Tanzania regarding mutations in *Pfcrt* (reviewed in [33]) and artemether-lumefantrine regarding the Pfmdr1 marker [48]. Furthermore, occurrence of the South Asian CQ-resistant *Pfcrt* haplotype SVMNT was also reported in Tanga for the first time [49].

Local drug pressure is mainly related to local malaria endemicity, which meaning that resistance levels may vary between different regions in Tanzania, due to their differences in malaria endemicity. The whole of Tanzania is endemic to malaria, but the Indian Ocean coastal strip (Tanga, Coastal, Dar es Salaam, Lindi, Mtwara) and the Lake Victoria shores (Mwanza, Kagera) have generally higher malaria endemicity compared to the rest of the country. The high malaria endemicity in, especially, Tanga and Kagera may therefore partly explain the observed high resistance to SP in these regions due to increased drug pressure relative to other regions. However, the differences in levels of resistance markers between these regions compared to the Coastal region, which also represents high malaria endemicity, leads to speculation that other local factors are contributing to local expansion of the 581G mutant parasites. Such factors may be related to local movement of the population and the in- and out-flow of people carrying parasites from one area to another. For instance, according to a study by Pindolia et al. [50], the Dodoma region is a major source of both human movement and malaria movement. Pindolia and colleagues have shown that while the majority of both human and malaria movement is directed towards the central and the western parts of the country, which do not represent major sources of super resistance (mainly Mbeya and Mwanza), the majority of the malaria movement was directed towards Mwanza (where there is some degree of resistance). This implies that the malaria movement does not correlate positively with resistance. Tanga, however, which represents the highest level of SP resistance was found to represent only internal movement and no major in- or out-flow or neither people nor malaria, indicating that perhaps this setting is more isolated and prone to resistance emergence and accumulation, perhaps due to a lack of in-flow of new and diverse parasite genotypes.

Furthermore, the southern parts of the Tanzania have a lower level of SP resistance, whereas the trends shown in the present study point to the northeastern and northwestern parts bordering Kenya and Uganda, respectively, having relatively higher levels of SP resistance. It is possible that cross-border spread of resistance contributes to these observations. For instance, in western Kenya, *Pfdhps* 540E and 581G have been reported to have increased, respectively, from 33.1 and 0 % in 1996 to 99.2 and 5.3 % in 2009 [43]. Related trends have also been reported in the same area in Kenya [51] although the reported prevalence of 581G is much lower than in Tanga and Kagera regions. In Uganda, a study performed in central Uganda reported *Pfdhps* 581G at 3.3 %, while another in western Uganda reported 36 % [28, 52].

Considering the combined *Pfdhps* K540E-A581G haplotypes, the EG haplotype (540E–581G) was observed at highest prevalence (>50 %) in Tanga region (both in Muheza and Bondo sites) and in Kagera (>20 % in Muleba). This haplotype is common in South America and Cambodia [53] thus it unclear whether this has independently evolved in Tanzania or other factors such as importation account for this. The EA highest in Mbeya and Mwanza regions (>90 %) was considered an East African haplotype, while the rarely observed wild-type KA is common in West Africa. A rare KG haplotype in was observed in Tanga. The KG is thought to evolve from the EG haplotype and is common in Cambodia and Venezuela [53]. The overall distribution of *Pfdhps* mutation and especially the EG haplotype indicates that *P. falciparum* sensitivity to SP vary largely across the country, with implication for SP-IPTp implementation. Despite the large sample set from across Tanzania used in this study, it is not clear if a greater variability can be found if further sampling is performed. Furthermore, intra-regional variability in resistance has been observed, for instance, for the case of Bondo and Muheza within Tanga region. Further preliminary mapping of the *Pfdhps* K540E-A581G haplotypes using 2014 samples from Songea in Ruvuma region, which is placed somewhat between Mtwara and Mbeya, observed a prevalence of the EG haplotype of 2 % (Fig. 1), which does not differ much from the 5 % in neighbouring Mtwara region. A similar observation was made for Mwanza.

Sporadic evidence shows that although ACT is the current first-line anti-malarial for treatment of uncomplicated malaria, the lack of proper healthcare services, especially in rural communities in Tanzania, as well as the availability of other anti-malarials in drug shops, encourages self-medication with non-standard regimens, including SP [54, 55]. It is important for national malaria control programmes and Ministries of Health to limit the availability of drugs such as SP to IPTp and/or IPTi programmes only. Furthermore, consideration for differentiated regional implementation of SP-IPTp based on differences in resistance levels may be important prior to availability of alternatives to SP. Sustained monitoring of molecular markers of SP resistance, especially super-resistance markers, should be continued with countrywide coverage in order to enable time-point specific assessment.

## Conclusions

Selection of SP resistance in Tanzania is high with variable distribution of *Pfdhps* A581G SP super-resistance marker across the country. The northeastern Tanga region and the northwest Mwanza and Kagera regions have high prevalence of SP super resistance that may spread to other regions of the country. This variable distribution of SP super resistance may have implications for the current IPTp policy and its outcome in Tanzania. Until alternative drugs for IPTp are available, routine monitoring of clinical outcome of IPTp and continuous monitoring of SP super-resistance markers is necessary.

## Abbreviations

ACT: artemisinin-based combinational therapy
CQ: chloroquine
SMC: seasonal malaria chemoprevention
ELISA: enzyme-linked immunosorbent assay
IPTi: intermittent preventive treatment of malaria in infancy
IPTp: intermittent preventive treatment of malaria in pregnancy
NIMR: National Institute of Medical Research
PCR–RFLP: polymerase chain reaction-restriction fragment length polymorphisms
Pdhfr: *Plasmodium falciparum* dihydrofolate reductase
Pfdhps: *Plasmodium falciparum* dihydropteroate synthetase
SP: sulfadoxine–pyrimethamine
SPSS: statistical package for social sciences
SSOP: single-strand oligonucleotide probes
THRiVE: Training Health Researchers into Vocational Excellence in East Africa
WHO: World Health Organization
